# The Acceptability of an Electronically Delivered Acceptance- and Mindfulness-Based Physical Activity Intervention for Survivors of Breast Cancer: One-Group Pretest-Posttest Design

**DOI:** 10.2196/31815

**Published:** 2022-04-29

**Authors:** Michael C Robertson, Emily Cox-Martin, Ross Shegog, Christine M Markham, Kayo Fujimoto, Casey P Durand, Abenaa Brewster, Elizabeth J Lyons, Yue Liao, Sara A Flores, Karen M Basen-Engquist

**Affiliations:** 1 Department of Nutrition, Metabolism, and Rehabilitation Sciences The University of Texas Medical Branch Galveston, TX United States; 2 Department of Behavioral Science University of Texas MD Anderson Cancer Center Houston, TX United States; 3 Health Promotion & Behavioral Sciences University of Texas School of Public Health Houston, TX United States; 4 VA Puget Sound Health Care System Tacoma, WA United States; 5 Department of Clinical Cancer Prevention University of Texas MD Anderson Cancer Center Houston, TX United States; 6 College of Nursing and Health Innovation The University of Texas Arlington, TX United States; 7 Department of Health and Kinesiology Texas A&M University College Station, TX United States

**Keywords:** cancer survivors, exercise, mindfulness, Acceptance and Commitment Therapy, behavioral sciences

## Abstract

**Background:**

Survivors of breast cancer can face internal barriers to physical activity, such as uncertainty and frustration stemming from physical limitations, decreased physical functioning, fatigue, and pain. Interventions that draw from the principles of Acceptance and Commitment Therapy (ACT) may help survivors of breast cancer overcome some of the internal barriers associated with physical activity.

**Objective:**

The primary aim of this study was to investigate the acceptability of an electronically delivered physical activity intervention for survivors of breast cancer, centered on ACT processes.

**Methods:**

This study used a 1-group pretest-posttest design. We recruited 80 insufficiently active female survivors of breast cancer using a web-based recruitment strategy. The 8-week intervention consisted of weekly modules that featured didactic lessons and experiential exercises targeting key ACT processes in the context of physical activity promotion (namely, values, committed action, acceptance, defusion, and contacting the present moment). We determined intervention acceptability according to study retention (≥70%), adherence rates (≥75% of the participants completing ≥50% of the modules), and posttest survey scores reflecting the perceived ease of use, perceived usefulness, and interest and enjoyment of the intervention (≥5 on a 7-point Likert-type scale). We also evaluated changes in self-reported aerobic and muscle strengthening–physical activity, physical activity acceptance, physical activity regulation, and health-related outcomes.

**Results:**

The retention rate (61/80, 76%), adherence rate (60/80, 75%), average perceived ease of use (6.17, SD 1.17), perceived usefulness (5.59, SD 1.40), and interest and enjoyment scores (5.43, SD 1.40) met the acceptability criteria. Participants increased their self-reported aerobic physical activity (Cohen *d*=1.04), muscle strengthening–physical activity (Cohen *d*=1.02), physical activity acceptance (cognitive acceptance: Cohen *d*=0.35; behavioral commitment: Cohen *d*=0.51), physical activity regulation (identified regulation: Cohen *d*=0.37; integrated regulation: Cohen *d*=0.66), increased their ability to participate in social roles and activities (Cohen *d*=0.18), and reported less fatigue (Cohen *d*=0.33) and sleep disturbance (Cohen *d*=0.53).

**Conclusions:**

Electronically delivered acceptance- and mindfulness-based interventions may be useful for promoting physical activity in survivors of breast cancer. Further research is needed to refine these approaches and evaluate their effectiveness.

## Introduction

### Background

Despite the well-documented benefits of physical activity, most survivors of breast cancer do not meet the nationally recommended physical activity guidelines [1,2]. This population may encounter challenges in meeting the recommended levels of physical activity common to the general US population, along with barriers attributable to cancer and its treatment. These can include uncertainty and frustration stemming from physical limitations, decreased physical functioning, fatigue, and pain associated with physical activity [3-6].

Behavioral interventions based on the principles of Acceptance and Commitment Therapy (ACT) may be useful in helping survivors of breast cancer increase physical activity. This is partly because many of the barriers to physical activity attributable to cancer and its treatment are internal in nature and are not necessarily amenable to immediate problem solving. ACT is an approach to behavioral therapy that supplements behavioral skill building with techniques centered on developing *psychological flexibility*: the ability to be aware of, accept, and proceed with gentle persistence despite uncomfortable sensations, thoughts, and feelings that may accompany behaviors consistent with personal values [7]. It encourages individuals to set goals and take committed action in the service of clearly defined values. Rather than identifying and seeking to change problematic thoughts, emotions, and physical sensations that can stand in the way of valued living, ACT focuses on changing how individuals relate to these thoughts and feelings. Compelling evidence demonstrates that ACT is effective in bringing about a broad range of psychological and behavioral outcomes [8,9] and has shown promise for helping cancer survivors cope with negative internal experiences that can accompany cancer diagnosis and treatment [10,11].

Although ACT is typically delivered face-to-face by trained mental health professionals in clinical settings, ACT principles and skills are increasingly being applied remotely to promote behavior change for public health priorities, such as smoking cessation, weight management, diabetes management, and physical activity [12-16]. A recent systematic review and meta-analysis concluded that interventions based on ACT principles hold promise for increasing physical activity, but their application to this end is nascent [17]. The degree to which this approach to physical activity promotion, delivered electronically, may be appropriate and useful for survivors of breast cancer is unknown.

### Objectives

The primary aim of this study was to investigate the acceptability of the ACTive program, an electronically delivered acceptance- and mindfulness-based physical activity intervention designed for survivors of breast cancer. This research corresponds to *phase IIa: Proof-of-Concept* of the Obesity-Related Behavioral Intervention Trials model for developing behavioral treatments [18]. It follows formative qualitative research [19] and systematic intervention development and refinement [20]. Our primary hypothesis was that female survivors of breast cancer exposed to the ACTive program would rate it as acceptable, as defined by study retention, program adherence, and ratings of perceived ease of use (PEOU), usefulness, and intrinsic motivation. Exploratory aims were to evaluate changes in participants’ physical activity, related cognition, and health-related outcomes associated with receiving the behavioral intervention.

## Methods

### Recruitment

Eligibility criteria included that the participants be female adults with a history of breast cancer diagnosis who were not undergoing chemotherapy or irradiation treatment and were not planning on or preparing for surgery. Furthermore, participants were not eligible for inclusion if upon eligibility screening their modified Physical Activity Readiness Questionnaire [21] score indicated that unsupervised physical activity may not be safe, or the modified Godin Leisure-Time Exercise Questionnaire [21] indicated that they tended to engage in ≥150 minutes of moderate intensity aerobic exercise per week (or ≥75 minutes of vigorous intensity aerobic exercise per week or an equivalent combination of physical activity volume).

We recruited participants using the services of the Love Research Army of the Dr Susan Love Research Foundation. The recruitment material was emailed to a large listserv consisting of approximately 79,000 individuals who had signed up to receive information about breast cancer–related research studies. Interested participants provided their contact information. The study staff contacted interested individuals via telephone to assess eligibility and engage in the informed consent process.

### Study Design

This study used a 1-group pretest-posttest design. Participants were recruited in September 2020 and completed a baseline survey about demographic information, physical activity levels, physical activity acceptance, physical activity regulation, and quality of life. The intervention content was delivered over the course of 8 weeks, starting in the last week of September 2020. All participants started the intervention simultaneously. A week after completing the intervention, participants completed a follow-up survey gathering information about the acceptability of the intervention, physical activity levels, physical activity acceptance, physical activity regulation, and quality of life. Surveys were delivered via REDCap (Research Electronic Data Capture; Vanderbilt University).

### Ethics Approval

All study procedures were approved by the University of Texas School of Public Health Committee for the Protection of Human Subjects (HSC-SPH-18-1025). All participants provided informed consent for participation before taking part in the study.

### Intervention Development

**Figure 1:**
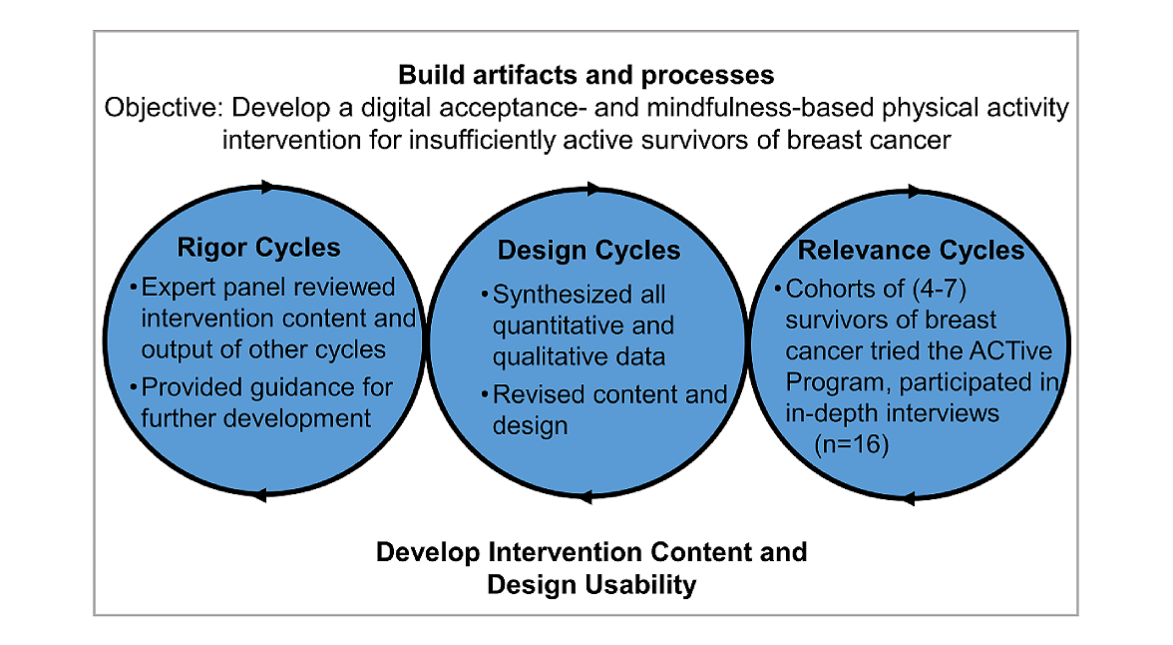
Information Systems Research iterative design framework for the intervention.

Before this study, we developed the ACTive program using an iterative design process. We used an existing manual to guide the application of ACT principles to help insufficiently active individuals increase physical activity [22]. To frame the intervention development process, we used the Information Systems Research framework [23]. This approach frames intervention development in three cycles (ie, *design*, *rigor*, and *relevance* cycles), which are iteratively repeated (Figure 1). Throughout this process, we included insights from individuals from the target population (30/80, 37% of the participants met the aforementioned eligibility criteria and were recruited using the same methods). The lead author (MCR) conducted individual interviews with participants after they experienced the development of intervention content and revised the intervention based on the findings from these interviews. The results of qualitative analyses are presented in the qualitative study by Robertson et al [20]. Throughout this process, we identified and iteratively tested the practical aspects of the ACTive program design. For example, we found REDCap to be an intervention delivery modality that could securely deliver intervention content (including potentially sensitive information) in a way that was perceived as simple and easy to navigate. Furthermore, we included mixed types of media (eg, short videos and audio files, images, text, and documents) and added components that participants requested, such as resources with instructions on how to safely engage in muscle strengthening–physical activity and gentle yoga classes.

### Intervention

The ACTive program (Textbox 1) [24] was designed to help insufficiently active survivors of breast cancer meet the 2018 aerobic- and muscle strengthening–physical activity guidelines for Americans according to their own physical activity–related preferences and abilities. Target guidelines included engaging in 150 minutes of moderate intensity aerobic physical activity per week (or 75 minutes of vigorous intensity aerobic physical activity per week or an equivalent combination of both exercise intensities), engaging in at least two bouts of muscle strengthening–physical activity that targeted all major muscle groups per week [25].

**Template for Intervention Description and Replication checklist for the present intervention.**

**The ACTive program briefs and description**

- Why?
  Despite the well-documented benefits, most survivors of breast cancer do not meet nationally recommended physical activity guidelines. Behavioral interventions based on the Acceptance and Commitment Therapy principles may be useful for helping survivors of breast cancer to increase physical activity. Digital behavior change interventions minimize barriers to access that can undermine traditional behavioral interventions.
- What (materials)?
  The ACTive program consisted of 9 modules that featured didactic lessons and experiential exercises targeting key Acceptance and Commitment Therapy processes (Table 1). In addition, the ACTive program featured cancer survivor–specific resources for engaging in aerobic- and muscle strengthening–physical activity and delivered behavior change techniques for safely increasing physical activity. See the Methods section for more details and references to external content.
- What (procedures)?
  The ACTive program was designed to help insufficiently active survivors of breast cancer gradually strive toward meeting the 2018 physical activity guidelines for Americans in accordance with their own physical activity–related preferences and abilities. The participants were sent intervention content weekly. They were encouraged to view all intervention content and provide responses to all queries before the next weekly module was sent.
- Who provided?
  All intervention content was created or curated by the principal investigator of the study (MCR), a doctoral student with an Master’s in Public Health studying behavioral science. See the Methods section for more details and references to external content.
- How?
  The intervention was delivered via REDCap (Research Electronic Data Capture). REDCap was also used to periodically send participants emails from the principal investigator’s (MCR) email address acknowledging the participants’ effort and responses (eg, providing participants with their statements of values, goals, and committed action).
- Where?
  The intervention content was delivered via the internet to participants throughout the United States.
- When and how much?
  The ACTive program was delivered over the course of 8 weeks, starting from the last week of September 2020. Per week, 1 module was sent (the first week additionally contained a brief Getting Started module).
- Tailoring:
  The participants were regularly reminded of their previous responses and were prompted to build upon them (eg, in week 3, participants were presented with the personal values they identified in week 2 and asked to set corresponding goals and engage in action planning). The intervention also provided optional resources, and individuals were encouraged to use those that they found to be personally relevant (eg, information pertaining to physical activity and lymphedema).
- How well?
  All intervention content was successfully sent to participants’ preferred email addresses. Study retention and intervention adherence are the end points detailed in the Results section of this paper.

**Table 1:** ACTive program module topics and featured behavior change techniques (BCTs).

Module	Main topic (with the Acceptance and Commitment Therapy processes)	BCTs for physical activity promotion^a^
1	Introductory session: introduces study staff; establishes expectations	Motivational interviewing (confidence ruler to elicit positive change talk); time management
2	The benefits of physical activity: relevant scientific literature on physical activity; ways to gauge intensity	Provide information on consequences of behavior *in general*; environmental restructuring; provide instructions on how to perform the behavior; demonstrate the behavior
3	Values: identifying and clarifying personal values; how adherence to physical activity may support these values; increasing motivation	Stress management and emotional control training; prompt self-monitoring of behavior; provide instructions on how to perform the behavior; demonstrate the behavior
4	Goals and committed action: identifying goals consistent with values, including at least one physical activity–related goal; taking committed action to accomplish goals; distinguishing internal and external barriers to physical activity	Stress management and emotional control training; prompt self-monitoring of behavior; goal setting (behavior); action planning; provide instructions on how to perform the behavior; demonstrate the behavior
5	Acceptance: increasing acceptance as it applies to distress tolerance and physical activity; discriminating between acknowledgment and avoidance of internal discomfort; also included a *creative hopelessness* exercise	Stress management and emotional control training; prompt self-monitoring of behavior; goal setting (behavior); set graded tasks; provide rewards contingent on successful behavior; barrier identification and problem solving^a^; provide instructions on how to perform the behavior; demonstrate the behavior
6	Cognitive defusion: breaking the link between thoughts and behavior; becoming more aware of thoughts that may interfere with exercise plans	Stress management and emotional control training; prompt self-monitoring of behavior; goal setting (behavior); set graded tasks; provide rewards contingent on successful behavior; barrier identification and problem solving^b^; provide instructions on how to perform the behavior; demonstrate the behavior
7	Mindfulness: contacting the present moment; being present; allowing negative internal events to pass without disrupting committed action; engaging in nonjudgmental contact with psychological and physical events that occur; increasing awareness during physical activity	Stress management and emotional control training; prompt self-monitoring of behavior; goal setting (behavior); set graded tasks; provide rewards contingent on successful behavior; provide instructions on how to perform the behavior; demonstrate the behavior
8	Review: review and integrate key concepts	Stress management and emotional control training; prompt self-monitoring of behavior; goal setting (behavior); set graded tasks; provide rewards contingent on successful behavior; provide instructions on how to perform the behavior; demonstrate the behavior
9	Maintenance: how to maintain adherence to physical activity; navigating lapses; preventing relapse	Plan social support or social change; relapse prevention and coping planning; stress management and emotional control training; prompt self-monitoring of behavior; provide rewards contingent on successful behavior; provide instructions on how to perform the behavior; demonstrate the behavior

^a^On the basis of the Michie taxonomy [26].

^b^Problem solving was applied to external problems that may be readily amenable to change, but acceptance was applied to internal problems that may be more resistant to short term changes.

The intervention consisted of 9 modules that featured didactic lessons and experiential exercises targeting key ACT processes (namely, values, committed action, acceptance, defusion, and contacting the present moment) in the context of physical activity promotion for cancer survivors (Table 1). Sessions began with a mindfulness exercise designed to focus participants’ attention in preparation for lesson content and foster the initiation of a mindfulness practice. Didactic lessons typically consisted of multiple 3- to 5-minute video and audio files narrated by the principal investigator of the study (MCR); these were supplemented by outside sources from ACT experts (eg, videos created by Dr Russ Harris [27,28]). Sessions also featured workbook-type activities and exercises designed to apply didactic content to their lives (eg, having participants identify their personally held values).

In addition to acceptance- and mindfulness-based content, the ACTive program featured resources for engaging in physical activity and applying commonly used behavior change techniques for physical activity promotion (Table 1) [26]. These resources included cancer survivor–specific how-to videos for engaging in muscle strengthening–physical activity (eg, embedded links to the Oncology, Nutrition and Exercise Group exercise videos by PennState [29], a video on proper walking posture by an exercise physiologist, and recorded yoga sessions for cancer survivors) as well as other audiovisual components (eg, images with supportive messages or inspirational quotes) and supporting documents (eg, a habit tracker and a printable calendar). The participants were prompted to report their weekly physical activity levels to facilitate self-monitoring. If participants (1) reported meeting the recommended guidelines for aerobic physical activity or muscle strengthening exercise, (2) met their own personally set physical activity–related goals, or (3) improved their aerobic physical activity from the week before, they were immediately rewarded with celebratory images and statements acknowledging the achievement (no additional content was added if participants did not meet any of these criteria).

The intervention was delivered in an automated fashion via REDCap, which sent surveys containing all intervention content through a dedicated study email address. To facilitate a sense of supportive accountability [30], REDCap was used to automatically send participants emails from the principal investigator’s (MCR) email address upon completion of various aspects of the intervention. These emails acknowledged participation and provided participants with their own responses for their records (eg, providing participants with their values, goals, and statements of committed action). Further, the REDCap surveys were programmed to automatically provide reminders of previous input responses so that participants could build upon them (eg, participants were presented with what they put as their values upon being prompted to engage in goal setting and action planning).

### Measures

#### Acceptability

Our conceptualization of the ACTive program’s *acceptability* was based on study retention and adherence rates and the Integrated Model of Technology Acceptance (IMTA). [31,32]. We calculated the ACTive program’s *retention* rate as the percentage of participants who completed the follow-up survey. We calculated the *adherence* rate from the percentage of modules completed by each participant as indicated by the REDCap system use data. IMTA is a measurement model for eHealth technology acceptance. It unifies previous lines of research of information systems acceptance and posits that technology adoption is best predicted by PEOU, perceived usefulness (PU), and intrinsic motivation [31,32]. To measure these constructs, we used the PEOU scale [31,32], the PU scale [31,32], and the interest/enjoyment subscale of the Intrinsic Motivation Inventory (IMIe) [33] (Table 2). The PEOU and PU scales consist of six 7-point Likert-type items (eg, “Learning to operate this intervention would be easy for me” and “I would find this intervention to be useful for being more physically active,” respectively), with responses ranging from *Extremely unlikely* to *Extremely likely*. A psychometric analysis of these scales found evidence of reliability (Cronbach α of .98 for PU and .94 for PEOU) and convergent, discriminant, and factorial validity [34]. The IMIe scale consists of seven 7-point Likert-type items (eg, “I enjoyed doing this activity very much”), with responses ranging from *1 (Not at all true)* to *7 (very true)*. This subscale has demonstrated good internal consistency and test-retest reliability in diverse populations [33,35,36].

**Table 2:** Summary of operationalizing measures.

Construct and component		Operationalization	Internal reliability^a^	Example item
**Acceptability**				
	Retention	Percentage of participants who completed the follow-up survey	N/A^b^	N/A
	Adherence	Percentage of modules completed	N/A	N/A
	Ease of use	Perceived ease of use scale [31,32]	.95	“Learning to operate this intervention would be easy for me.”
	Usefulness	Perceived usefulness scale [31,32]	.97	“I would find this intervention to be useful for being more physically active.”
	Enjoyability	Interest and enjoyment subscale of the Intrinsic Motivation Inventory [33]	.92	“I enjoyed doing this activity very much.”
**Physical activity**				
	Leisure-time aerobic physical activity	Godin Leisure-Time Exercise Questionnaire [37]	N/A	“During a typical 7-d period (a week), how many times on average do you do the following kinds of exercise for more than 15 minutes during your free time? Moderate Exercise (not exhausting; eg, fast walking, baseball, tennis, easy bicycling, volleyball, badminton, easy swimming, alpine skiing, popular and folk dancing).”
	Muscle strengthening–physical activity	Modified Godin Leisure-Time Exercise Questionnaire [38,39]	N/A	“In a typical week, outside of your job or work around the house, how many days do you do leisure-time physical activities specifically designed to strengthen your muscles such as lifting weights, circuit training, or resistance bands? (Do not include cardio/aerobic types of exercise).”
**Physical activity acceptance**				
	Cognitive acceptance	Cognitive acceptance subscale of PAAQ^c^	.75	“I need to concentrate on getting rid of my urges to stop exercising or put off exercise.”
	Behavioral commitment	Behavioral commitment subscale of PAAQ	.81	“I am committing to being physically active no matter what feels uncomfortable or challenging about that.”
**Physical activity motivation**				
	Amotivation	Amotivation subscale of BREQ-3^d^	.84	“I don’t see why I should have to exercise.”
	External regulation	External regulation subscale of BREQ-3	.86	“I exercise because other people say I should.”
	Introjected regulation	Introjected regulation subscale of BREQ-3	.84	“I feel guilty when I don’t exercise.”
	Identified regulation	Identified regulation subscale of BREQ-3	.79	“It’s important to me to exercise regularly.”
	Integrated regulation	Integrated regulation subscale of BREQ-3	.88	“I exercise because it is consistent with my life goals.”
	Intrinsic regulation	Intrinsic regulation subscale of BREQ-3	.93	“I exercise because it’s fun.”
**Health-related outcomes**				
	Physical function	Physical function subscale of PROMIS-29^e^	.78	“Are you able to do chores such as vacuuming or yard work?”
	Anxiety	Anxiety subscale of PROMIS-29	.89	“In the past 7 days...I felt fearful.”
	Depressive symptoms	Depressive symptoms subscale of PROMIS-29	.87	“In the past 7 days...I felt worthless.”
	Fatigue	Fatigue subscale of PROMIS-29	.94	“In the past 7 days...how run-down did you feel on average?”
	Sleep disturbance	Sleep disturbance subscale of PROMIS-29	.88	“In the past 7 days...I had difficulty falling asleep...”
	Ability to participate in social roles and activities	Ability to participate in social roles and activities subscale of PROMIS-29	.90	“I have trouble doing all of the activities with friends that I want to.”
	Pain interference	Pain interference subscale of PROMIS-29	.94	“In the past 7 days...how much did pain interfere with your day to day activities?”

^a^Cronbach *α* at follow-up of this study.

^b^N/A: not applicable.

^c^PAAQ: Physical Activity Acceptance Questionnaire [40].

^d^BREQ-3: Behavioral Regulation for Exercise Questionnaire-3 [41].

^e^PROMIS-29: Patient-Reported Outcomes Measurement Information System-29 profile measure (version 2.1) [42].

#### Physical Activity

To assess physical activity levels, the Godin Leisure-Time Exercise Questionnaire was administered. This questionnaire has been shown to have good retest reliability (reliability coefficient=0.81) and convergent validity with measures of fitness such as maximum rate of oxygen consumption during intense exercise [37] and has been identified as a useful measure for understanding physical activity patterns in survivors of breast cancer [43]. We modified the Godin Leisure-Time Exercise Questionnaire to add an item measuring muscle strengthening–physical activity as has been done elsewhere in populations of cancer survivors [38,39]. This item reads, “In a typical week, outside of your job or work around the house, how many days do you do leisure-time physical activities specifically designed to strengthen your muscles such as lifting weights, circuit training, or resistance bands? (Do not include cardio/aerobic types of exercise)” and response options ranged from 0 to 7.

#### Physical Activity Acceptance

A central construct targeted by the ACTive program is experiential acceptance, defined as the propensity to acknowledge negative internal experiences rather than avoid them. We operationalized this construct using the Physical Activity Acceptance Questionnaire (PAAQ) [40]. This questionnaire consists of two subscales, *cognitive acceptance* (eg, “I need to concentrate on getting rid of my urges to stop exercising or put off exercise”) and *behavioral commitment* (eg, “I am committing to being physically active no matter what feels uncomfortable or challenging about that.”). Responses ranged from *1 (Never true)* to *7 (Always true)*. This questionnaire has demonstrated sound psychometric properties in survivors of breast cancer, with high internal validity (Cronbach *α*=.89), test-retest reliability, and convergent validity with established measures of mindfulness and physical activity (both self-reported and accelerometer-measured) [40].

#### Physical Activity Motivation

A recent meta-analysis and systematic review revealed that mindfulness can have marked effects on motivation for health-related behaviors (as conceptualized by Self-Determination Theory) [44]. To investigate this link in the context of this study, we evaluated the participants’ physical activity–related motivation at baseline and after the intervention. To do so, we administered the 24-item Behavioral Regulation for Exercise Questionnaire-3 (BREQ-3) [41]. This questionnaire contains 5 subscales that operationalize Self-Determination Theory constructs of *amotivation*, *external regulation*, *introjected regulation*, *identified regulation*, *integrated regulation*, and *intrinsic regulation* (eg, “It’s important to me to exercise regularly”). Responses ranged from *0 (Not true for me)* to *4 (very true for me)*. This questionnaire was found to have acceptable internal consistency in a sample of 414 survivors of colorectal cancer [45].

#### Health-Related Outcomes

To measure quality of life and physical functioning, we administered the Patient-Reported Outcomes Measurement Information System-29 profile measure (version 2.1; PROMIS-29) [42]. The PROMIS initiative is a National Institutes of Health initiative that aims to create psychometrically sound self-report measures designed to assess well-being in various domains of human health [46]. PROMIS-29 includes eight subscales, seven of which (physical function, anxiety, depressive symptoms, fatigue, sleep disturbance, ability to participate in social roles and activities, and pain interference) have 4 items with 5 Likert-type responses each (eg, ranging from *Not at all* to v*ery much*). The final subscale (pain intensity) has 1 item with responses ranging from *0 (No Pain)* to *10 (Worst pain imaginable)*. Scores were coded and summed such that higher scores indicate more of the concept being measured (ie, higher scores for physical function are favorable, but higher scores for anxiety are not favorable). Raw scores were then converted to *T*-scores using standardized PROMIS tables [42], which were rescaled such that the mean was 50 and the SD was 10. This questionnaire has demonstrated strong psychometric properties across a variety of populations, including cancer survivors [42,47-49].

#### Data Analysis

We computed participants’ average PEOU, PU, and IMIe scores in accordance with their recommended scoring procedures. We calculated the average weekly moderate to vigorous physical activity using the Godin Leisure-Time Exercise Questionnaire [37] and the average subscale scores for the PAAQ and BREQ-3, following the scoring instructions. We followed the recommended PROMIS procedures to calculate the *T*-score metrics from the participant responses. We used listwise deletion to handle missing data, which assumes that missing data are completely missing at random [50]. We set the nominal *α* to .05 and used R (version 4.0.3) [51] and the tidyverse package [52] to conduct the data analysis.

Following the CONSORT (Consolidated Standards of Reporting Trials) guidelines [53], we determined the a priori criteria upon which to base our decision regarding the acceptability of the ACTive program. These were based on retention rate, adherence rate, and IMTA-based acceptability questionnaire data. As has been done elsewhere, we set the criteria for an acceptable *retention rate* of ≥70% [54,55]. Our criterion for the *adherence rate* was that ≥75% of participants completed at least four of the modules, which is comparable with other digital behavior change interventions (DBCIs) for cancer survivors [55-57]. Finally, our acceptability criteria included that the average scores of PEOU, PU, and IMIe were ≥5 (out of the 7 points of the Likert-type scales) [58]. To pursue exploratory aims, we conducted 2-tailed, paired sample *t* tests (or paired sample Wilcoxon signed-rank test, as appropriate) and computed Cohen effect size values [59] for pre- and postintervention Godin Leisure-Time Exercise Questionnaire, PAAQ, BREQ-3, and PROMIS-29 subscale scores.

## Results

### Overview

We attempted to contact 134 participants who expressed interest in the study and met the prescreening eligibility criteria. Of the 134 participants, a total of 91 (67.9%) participants were formally screened. Of the 91 participants, 9 (10%) were found not eligible to participate (in most cases, because they were taking drugs for a heart condition), and 2 (2%) were found to be eligible but did not subsequently take part in the study. We engaged in an informed consent process with 90% (82/91) of participants, all of whom agreed to participate in the study. Of these 82 participants, 2 (2%) did not complete the baseline survey or receive any intervention content. Thus, 80 participants were included in the study’s analytic sample.

### Participant Characteristics

The mean age of the sample was 57.5 (SD 11.4, range 31-79) years, and the median time since breast cancer diagnosis was 7 (IQR 3-12) years. The study sample was relatively well-educated (64/80, 80% college graduates), mostly non-Hispanic White (58/80, 73%), and mostly either overweight or obese (58/79, 73%; Table 3).

**Table 3:** Participant characteristics (N=80).

Characteristic and category			Values, n (%)
**Education level**			
	Some college	16 (20)	
	Bachelor’s degree	36 (45)	
	Graduate school degree	28 (35)	
**Employment status**			
	Employed full time	41 (51)	
	Employed part-time	9 (11)	
	Retired	20 (25)	
	Other	10 (13)	
**Marital status**			
	Single	12 (15)	
	Married	58 (73)	
	Living with significant other	1 (1)	
	Divorced	5 (6)	
	Widowed	3 (4)	
**Race**			
	American Indian, Alaska Native, or other	1 (1)	
	Asian	4 (5)	
	Black or African American	7 (9)	
	White	65 (83)	
**Ethnicity**			
	Hispanic	7 (9)	
	Non-Hispanic	72 (91)	
**Stage of breast cancer at diagnosis**			
	1	33 (44)	
	2	30 (40)	
	3	10 (13)	
	4	2 (3)	
**BMI status**			
	Underweight	1 (1)	
	Normal	20 (25)	
	Overweight	34 (43)	
	Obese	24 (30)	

### Acceptability

**Figure 2:**
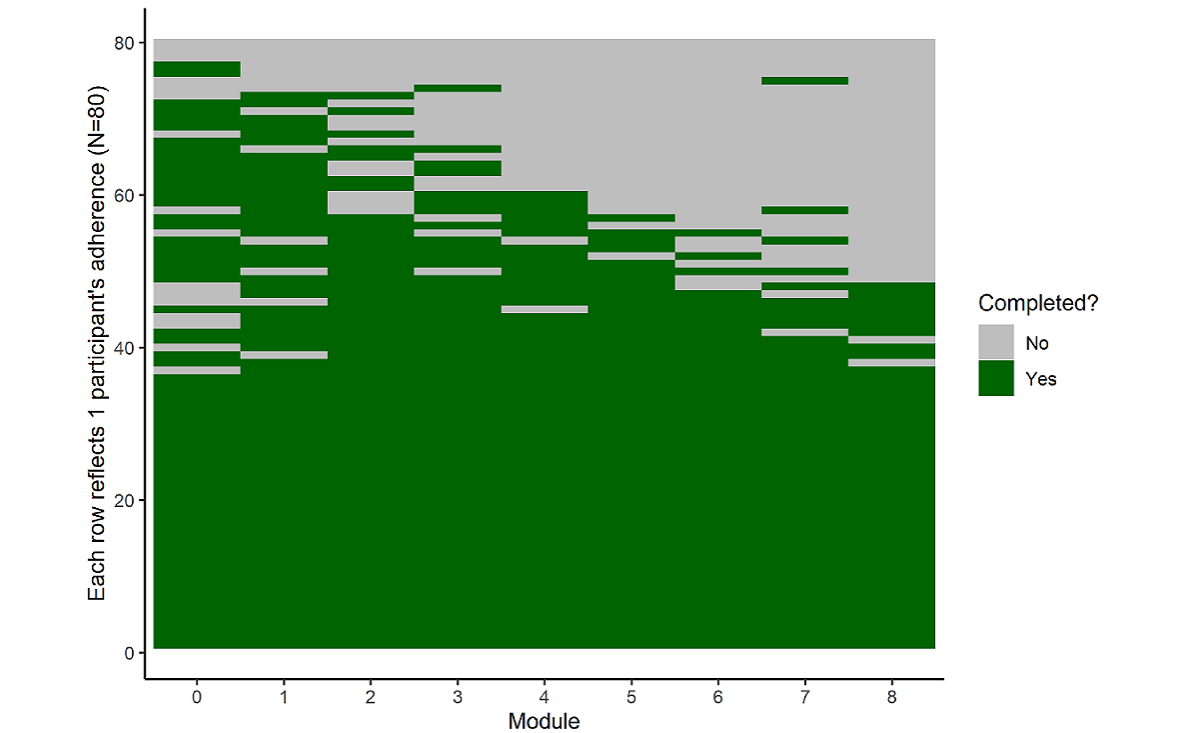
Participant completion of intervention modules.

**Figure 3:**
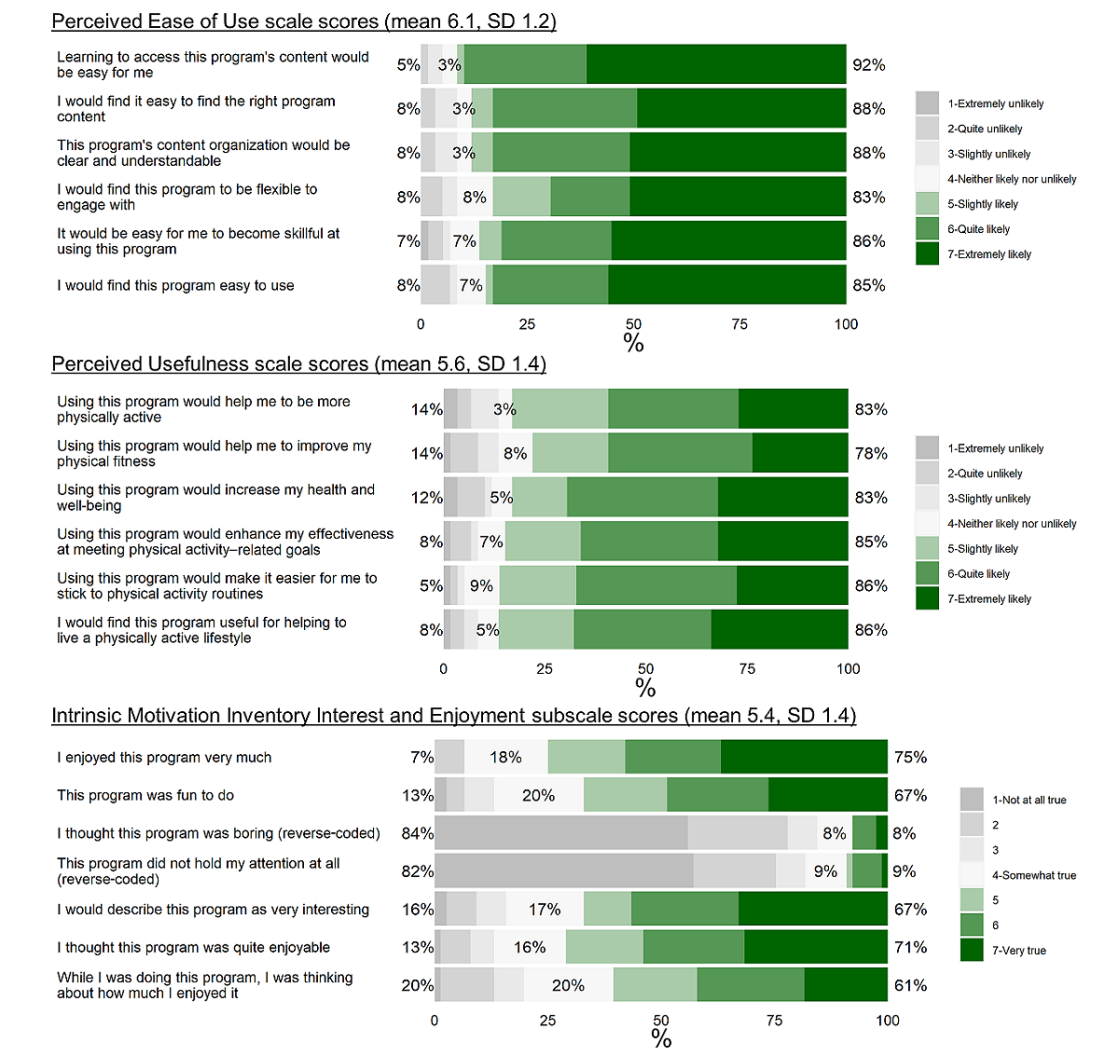
Acceptability scores. Inconsistencies in the sum of percentages is due to the rounding of the percentages.

Of the 80 participants in the analytic sample, 61 (76%) completed the follow-up survey after the 8-week intervention, yielding a *retention rate* of 76.3%. The participants completed 71.5% of all modules in total, and the *adherence rate* (percentage of participants who completed at least 4 modules) was 75% (60/80; Figure 2). The participants’ average PEOU, PU, and IMIe scores were 6.17 (SD 1.17), 5.59 (SD 1.40), and 5.43 (SD 1.40), respectively (Figure 3). The retention rate, adherence rate, and IMTA-based acceptability scores met the predetermined acceptability criteria.

### Exploratory Outcomes

**Figure 4:**
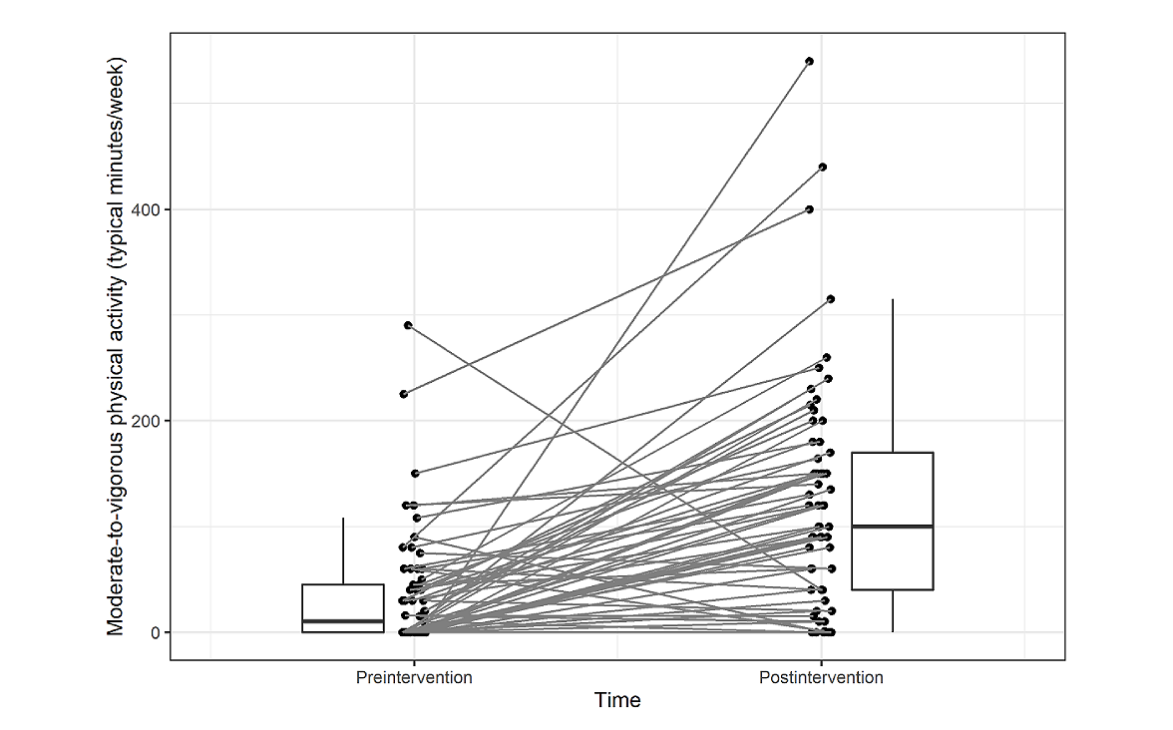
Pre- to postintervention change in average weekly moderate to vigorous physical activity as measured by the Godin Leisure-Time Physical Activity Questionnaire.

**Figure 5:**
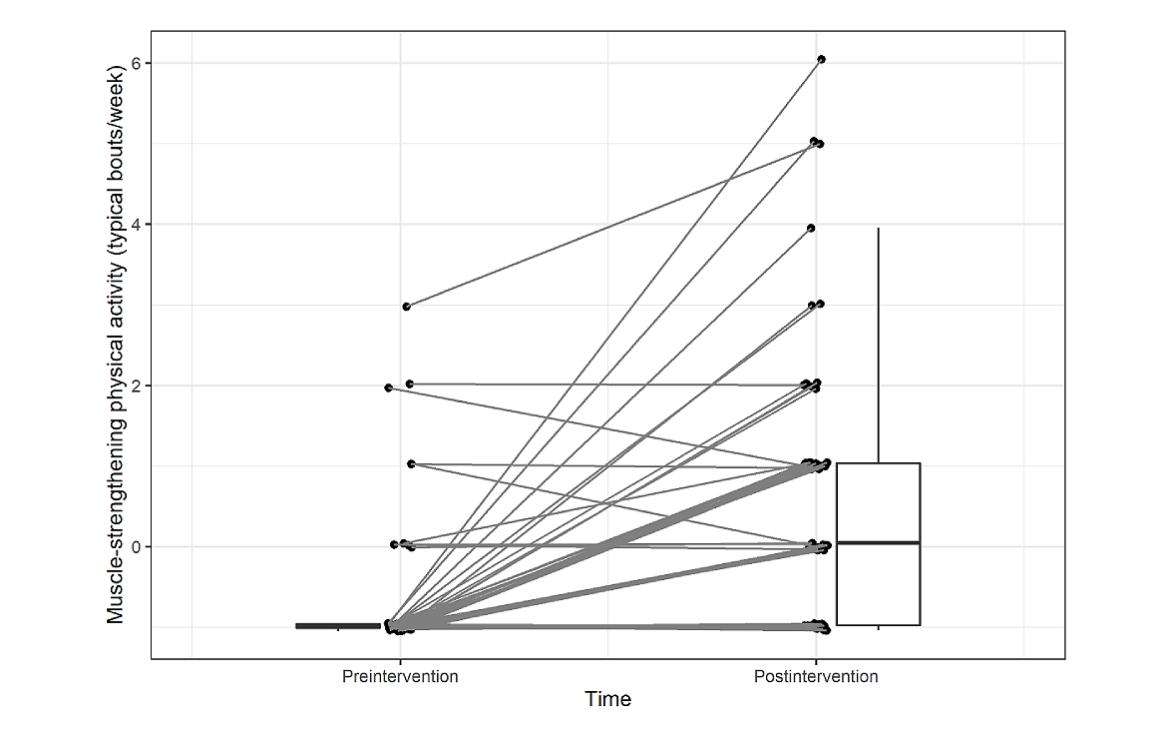
Pre- to postintervention changes in the average number of days participants engaged in muscle strengthening–physical activity as measured by the modified Godin Leisure-Time Physical Activity Questionnaire.

Table 4 presents the results of the exploratory analyses. On average, participating in the ACTive program was associated with an increase in nearly 90 minutes of self-reported moderate to vigorous intensity aerobic physical activity per week (Cohen *d*=1.04; Table 4; Figure 4) and 1.3 additional bouts of muscle strengthening–physical activity per week (Cohen *d*=1.02; Table 4; Figure 5). The participants exhibited statistically significant increases in scores for both the *cognitive acceptance* (Cohen *d*=0.35) and *behavioral commitment* subscales (Cohen *d=*0.51) of the PAAQ as well as for the *identified regulation* (Cohen *d=*0.37) and *integrated regulation* (Cohen *d=*0.66) subscales of the BREQ-3. There was no statistically significant increase in the *intrinsic regulation* subscale of the BREQ-3. Finally, participants exhibited decreased PROMIS-29 scores for *fatigue* (Cohen *d*=−0.33) and *sleep disturbance* (Cohen *d*=−0.53), and increased scores for ability to participate in social roles and activities (Cohen *d*=0.18) over the course of the study. The changes in the other PROMIS-29 subscales were not statistically significant (Table 4).

**Table 4:** Changes in exploratory outcomes associated with the ACTive program (n=59).

Questionnaire and construct or subscale		Baseline score, mean (SD)	Follow-up score, mean (SD)	Change, mean (SD)	*P* value
**Godin Leisure-Time Physical Activity Questionnaire**					
	Average weekly minutes of moderate to vigorous aerobic physical activity	36.2 (69.2)	127.4 (111.1)	91.6 (114.1)	<.001^a^
	Average weekly bouts of muscle strengthening–physical activity	0.3 (0.8)	1.6 (1.6)	1.3 (1.6)	<.001^a^
**Physical Activity Acceptance Questionnaire**					
	Cognitive acceptance	18.9 (6.9)	20.4 (6.0)	2.3 (6.9)	.01^b^
	Behavioral commitment	21.3 (5.5)	23.8 (4.7)	2.5 (5.2)	<.001^a^
**Behavioral Regulation for Exercise Questionnaire-3**					
	Identified regulation	2.5 (0.9)	2.8 (0.8)	0.3 (0.6)	<.001^a^
	Integrated regulation	1.5 (1.1)	2.1 (1.0)	0.7 (0.9)	<.001^a^
	Intrinsic regulation	1.7 (1.0)	1.9 (1.1)	0.2 (0.9)	.07^b^
**Patient-Reported Outcomes Measurement Information System-29 profile measure (version 2.1; *T*-scores)**					
	Physical function	53.1 (6.4)	53.3 (5.6)	0.2 (7.0)	.95^a^
	Anxiety	54.5 (9.1)	52.9 (8.6)	−0.6 (7.4)	.51^b^
	Depressive symptoms	51.1 (7.0)	49.8 (7.1)	−1.2 (5.6)	.11^a^
	Fatigue	53.3 (8.6)	50.2 (8.9)	−2.9 (9.2)	.02^b^
	Sleep disturbance	53.0 (7.8)	48.8 (8.0)	−4.2 (7.1)	<.001^b^
	Ability to participate in social roles and activities	52.1 (7.5)	53.5 (7.3)	1.3 (5.5)	.03^a^
	Pain interference	49.7 (7.8)	50.2 (8.2)	0.5 (8.1)	.69^a^
	Pain intensity (raw score)	3.5 (2.0)	3.5 (1.9)	0.08 (2.1)	.60^a^

^a^Paired-sample Wilcoxon signed-rank test.

^b^2-tailed, paired sample *t* test.

## Discussion

### Principal Findings

In this study, we evaluated the acceptability of the ACTive program, an acceptance- and mindfulness-based physical activity DBCI for insufficiently active survivors of breast cancer. The 8-week electronically delivered intervention was centered on the application of ACT principles to increase psychological flexibility and acceptance in the context of physical activity. The study retention rate; participant adherence rate; and PEOU, PU, and IMIe scores supported the acceptability of this approach for promoting physical activity in survivors of breast cancer. Exploratory findings suggest that participation in the program was associated with increased aerobic- and muscle strengthening–physical activity, physical activity acceptance, identified and integrated regulation of physical activity, and decreased fatigue and sleep disturbance.

Although it met the threshold we determined to be acceptable for this early phase of research, the retention rate for this study (74%) was relatively low. High attrition is a challenge commonly encountered in physical activity–related DBCIs [60], but our retention rate was modestly lower than some other studies in cancer survivors [61] and the general population [62]. In addition to extraneous factors such as the possibility of reduced participation because of the COVID-19 pandemic [63], this may be in part because of a relatively high participant burden. Full participation in the ACT-derived content featured in this study required a considerable degree of concentration and reflective thought. It may have been that participants who were lost to follow-up were not able to do so because of competing demands for time and energy. Future studies should investigate which subpopulations of survivors of breast cancer are most amenable to this unique approach to promote physical activity. Adaptive interventions may feature acceptance- and mindfulness-based modules for those who may benefit from this content the most.

Study adherence, operationalized in this study as the completion of the weekly modules, was relatively high. This is another known challenge to remotely deliver digital health studies; the participants commonly cease interacting with DBCI-related content in health-related studies in less than a week [64]. In this study, participation was close to 100% for approximately half of the participants (48/80, 60%) and gradually tapered off over time for the other half (32/80, 40%; Figure 2). Evidence suggests that physician referral is associated with markedly increased adherence to digital health studies and may be a way to improve adherence to empirically supported DBCIs [64]. Physical activity–related DBCIs may be a useful tool to supplement health care providers’ physical activity counseling, which has been shown to be effective but is often limited by time constraints [65]. Although the default assumption may be that more interactions with DBCI content is necessarily better, there is increasing recognition of the importance of parsing from intervention interaction that might constitute *effective engagement* or the level and type of engagement that is linked to key outcomes of interest [66]. The ACTive program was structured such that each module generally targeted specific ACT processes. It may be that some processes should be prioritized in the context of physical activity promotion if they predict a disproportionate amount of variance in physical activity–related outcomes. Future studies designed to evaluate intervention effectiveness should investigate what constitutes effective engagement with physical activity interventions centered on ACT principles. Furthermore, it may be useful to investigate the optimal constitution of ACT-based programs for promoting physical activity.

Findings pertaining to PEOU, PU, and IMIe scores indicated that the ACTive program was well received. These constructs predict the use and appraisal of web-based learning platforms [34,67-69] and the likelihood of cancer survivors sharing health-related information with others [70]. In this study, PEOU scores were particularly high (Figure 3). This finding supports the delivery of ACT-derived content to promote physical activity via digital means. This is a noteworthy finding, because to date, most physical activity interventions derived from ACT concepts have been conducted in person [17]. The findings suggest that this approach to physical activity promotion may be extended using DBCI technologies to increase public health impact. In this study, we used the REDCap survey delivery system. Although audiovisual program delivery is not its primary purpose, it seems to be useful for developing and evaluating beginning stage behavioral interventions. Furthermore, this may be a particularly attractive option when privacy and data security are paramount.

High PU and IMIe scores suggest that participating survivors of breast cancer felt that the application of acceptance- and mindfulness-based techniques to increase physical activity was relevant and enjoyable. This is an important finding given the marked heterogeneity of motivations for physical activity, physical abilities, and the range of desired DBCI features found in survivors of breast cancer [71]. This study is among the first to evaluate the use of acceptance- and mindfulness-based techniques for physical activity promotion in cancer survivors; although, ACT is increasingly being used to inform physical activity promotion interventions in other groups [17] and has been recommended as a useful therapeutic modality for cancer survivors [10,11]. The paradigm shifting emphasis to *change your relationship* with problematic thoughts and feelings, rather than changing the thoughts and feelings themselves, appears to resonate with insufficiently active survivors of breast cancer. High ratings of the PU of the intervention suggest that participants felt the program was effective at increasing their physical activity levels, and this notion was supported by exploratory findings.

The study participants tended to report substantial increases in aerobic- and muscle strengthening–physical activity levels from before the intervention to after the intervention. The participants averaged approximately 90 minutes per week increases in moderate to vigorous intensity aerobic physical activity and an approximately 1.3 bouts per week increase in muscle strengthening–physical activity. Given the dose response, negative association between physical activity and overall and cancer-specific mortality in survivors of breast cancer [72-75] and recommended guidelines for cancer survivors [76-78], these increases are clinically meaningful. The results are in accordance with a recent systematic review and meta-analysis that concluded that interventions based on ACT principles hold promise for increasing physical activity [17] and are supported by both high PU ratings and corresponding increases in PAAQ scores. Given the importance of long-term adherence to physical activity, future research is needed to evaluate the effectiveness of acceptance- and mindfulness-based interventions for both initiation and long-term maintenance of physical activity in survivors of breast cancer.

We observed small and medium effect sizes for changes in the PAAQ subscales of *cognitive acceptance* and *behavioral commitment*, respectively. This suggests that the participants experienced increases in both their experiential acceptance of physical activity–related internal experiences (eg, sensations, cognitions, and emotions) and their behavioral commitment to engaging in physical activity. This has implications for long-term change; increases in *cognitive acceptance* have been found to be associated with long-term changes in objectively measured physical activity [40]. As ACT is centered on increasing *psychological flexibility*, and in the context of physical activity promotion, this is perhaps most clearly manifested as *physical activity acceptance*, it may be that effective physical activity interventions derived from ACT tenets are partly mediated by this construct. Future studies should investigate this possibility in survivors of breast cancer.

Participants tended to report an increase in both *identified regulation* and *integrated regulation* of physical activity from before the intervention to after the intervention. These constructs are held by Self-Determination Theory to reflect autonomous forms of extrinsic regulation and have been shown to be consistently predictive of physical activity [79]. The findings of this study are concordant with the literature that has found mindfulness interventions to be associated with increases in autonomous motivation [44]. Practicing mindfulness exercises, such as engaging in mindful walking, might be theorized to increase the interest or enjoyment derived from physical activity and thus, engender increases in *intrinsic regulation* [44]. As changes in this study were observed for *identified regulation* and *integrated regulation* for physical activity but not *for intrinsic regulation*, it may have been that participants’ reflection on the benefits of physical activity alongside value clarification exercises caused them to value physical activity more deeply and increasingly identify as someone who prioritizes it. Future research should investigate this notion and how Self-Determination Theory and ACT may inform behavior change interventions in tandem.

Finally, sleep disturbance, fatigue, and the ability to participate in social roles and activities are challenges faced by cancer survivors that can begin with primary treatment and persist long into survivorship [80-82]. In this study, participants tended to report clinically meaningful decreases in these issues from before the intervention to after the intervention [83]. This finding is in accordance with the literature that has found effective physical activity interventions to impact these health-related outcomes in cancer survivors [84,85]. Indeed, the American College of Sports Medicine guidelines for cancer survivors provide specific physical activity recommendations for achieving improvements in these domains [77], and such changes may occur relatively quickly with increasing physical activity levels [86,87]. Other mean changes in health-related outcomes were not statistically significant; although, there were trends toward a reduction in depressive symptoms. However, the interpretation of changes in PROMIS-29 health-related needs to be considered in light of the COVID-19 pandemic and its societal ramifications, which may have influenced these variables.

### Strengths and Limitations

The findings of this study must be considered in the context of its limitations. The generalizability of this study is limited by convenience sampling methods that yielded a relatively well-educated sample and limited diversity in terms of race and ethnicity. Furthermore, participants who responded to the recruitment material may have been particularly motivated to increase their physical activity. The COVID-19 pandemic precluded more active forms of recruitment that may have yielded a more diverse sample, but our recruitment methods allowed individuals from all over the United States to participate. The study’s high attrition rate has potential implications for the findings regarding the acceptability of the intervention. It may have been that those who were lost to follow-up produced lower ratings. However, the results met the a priori criteria for determining the acceptability. Our study design was centered on investigating the acceptability of the ACTive program and precluded making causal inferences regarding the efficacy of the intervention. We observed that changes in reported physical activity along with high ratings of PU of the intervention and concomitant changes in theorized determinants and outcomes linked to physical activity are somewhat encouraging, but alternate explanations may account for these observations. Salient threats to internal validity include history (particularly given the COVID-19 pandemic), potential reactivity to the experimental situation, regression to the mean, and self-reported assessment of physical activity (which is prone to social desirability and recall bias). There is also an inflated chance of type 1 error given that we conducted multiple statistical tests (eg, evaluating changes in all survey subscales individually). We did not adjust the *P* values given the exploratory nature of this investigation. The strengths of this study include the use of a theory-based intervention that can be implemented with high fidelity and has potential for scalability, acceptability testing informed by the Obesity-Related Behavioral Intervention Trials model for intervention development, and predetermined thresholds to ascertain intervention acceptability. Another strength of this project was the parsimony of design and low cost of the intervention. The study was conducted with minimal resource expenditure using in-house scripting or video and leveraging extant resources (eg, REDCap). This low-end development was used to achieve considerable positive impact and demonstrated the ability to compile meaningful, theory-based applications for increased reach, fidelity, and acceptability.

### Conclusions

We conclude that electronically delivered acceptance- and mindfulness-based physical activity approaches to physical activity promotion represent potentially well-received and useful intervention option for insufficiently active survivors of breast cancer. Metrics pertaining to study retention, program adherence, and ratings of PEOU, usefulness, and intrinsic motivation all met the predetermined criteria for success. Receipt of the intervention was associated with increases in reported aerobic- and muscle strengthening–physical activity, physical activity acceptance, identified and integrated regulation of physical activity, and decreases in fatigue and sleep disturbance. More research is needed to further develop this approach to promote physical activity and formally evaluate its potential efficacy in pilot-testing with randomized designs.

## Abbreviations

ACT: Acceptance and Commitment Therapy
BREQ-3: Behavioral Regulation for Exercise Questionnaire-3
CONSORT: Consolidated Standards of Reporting Trials
DBCI: digital behavior change intervention
IMIe: interest/enjoyment subscale of the Intrinsic Motivation Inventory
IMTA: Integrated Model of Technology Acceptance
PAAQ: Physical Activity Acceptance Questionnaire
PEOU: perceived ease of use
PROMIS-29: Patient-Reported Outcomes Measurement Information System-29 profile measure (version 2.1)
PU: perceived usefulness
REDCap: Research Electronic Data Capture
